# Bioactivity‐guided isolation of anti‐inflammatory limonins from *Chukrasia tabularis*

**DOI:** 10.1002/fsn3.3015

**Published:** 2022-08-09

**Authors:** Jin‐Huang Shen, Yi‐Fan Zhang, Li Zhang, Na‐Na Yang, Xin‐Hua Ma, Tian‐Hua Zhong, Yong‐Hong Zhang

**Affiliations:** ^1^ Fujian Key Laboratory of Natural Medicine Pharmacology, School of Pharmacy Fujian Medical University Fuzhou China; ^2^ Medical Imaging Department First Affiliated Hospital of Fujian Medical University Fuzhou China; ^3^ Key Laboratory of Marine Biogenetic Resources, Third Institute of Oceanography Ministry of Natural Resources Xiamen China

**Keywords:** anti‐inflammation, *Chukrasia tabularis*, limonins, NO, TNF‐*α*

## Abstract

*Chukrasia tabularis* is an economically important tree and widely cultured in the southeast of China. Its barks, leaves, and fruits are consumed as a traditional medicine and perceived as a valuable source for bioactive limonin compounds. The extracts from root barks of *C. tabularis* showed significant anti‐inflammatory effect. The aim of this research was to explore the material basis of *C. tabularis* anti‐inflammatory activity, and to purify and identify anti‐inflammatory active ingredients. By a bioassay‐guided isolation of dichloromethane fraction obtained two novel phragmalin limonins, Chukrasitin D and E (**1** and **2**), together with 12 known limonins (**3**–**14**). The chemical structure of these compounds is determined on the basis of extensive spectral analysis and chemical reactivity. In addition, the activities of these isolated limonins on the production of nitric oxide (NO), tumor necrosis factor alpha (TNF‐α), and nuclear factor kappa B (NF‐*κ*B) in RAW264.7 cells induced by lipopolysaccharide (LPS) were evaluated. Limonins **1** and **2** indicated significant anti‐inflammatory activity with IC_50_ values of 6.24 and 6.13 *μ*M. Compound **1** notably inhibited the production of NF‐*κ*B, TNF‐*α* and interleukin 6 (IL‐6) in macrophages*.* The present results suggest that the root barks of *C. tabularis* exhibited anti‐inflammatory effect and the limonins may be responsible for this activity.

## Graphical abstract

Isolating limonins from Chukrasia tabularis including two new limonins and twelve known limonins. The limonins showed significant anti‐inflammatory activity in LPS‐induced RAW 264.7 cells. Compound 1 notably inhibited the production of NF‐κB, TNF‐α and IL‐6 in macrophages.

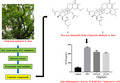

## 1 INTRODUCTION

Meliaceae plants are well known for their important biological activities and diversified limonoid compounds, and they have aroused widespread interest in organic chemistry and agricultural chemistry. The genus *Chukrasia* (Meliaceae) includes one species, *Chukrasia tabularis*, and a variant *Chukrasia abularis var. velutina* (Kaur & Arora, 2009; Liao et al., 2009; Mulholland et al., 2000; Roy & Saraf, 2006). In recent years, a large amount of limonins with diverse structures have been separated from this genus (Fan et al., 2007; Zhang, Fan, et al., 2008; Zhang, Yang, et al., 2008), some of them have anti‐inflammatory, potassium channel blocking, and antibacterial activity (Zhang, Fan, et al., 2008; Zhang, Yang, et al., 2008; Zhang, Yang, Liao, et al., 2007; Zhang, Yang, Zhu, et al., 2007). Phragmalin limonoids and carbonate are characteristic compositions of the *Chukrasia* genus (Abdelgaleil et al., 2006; Nakatani et al., 2004; Saad et al., 2003; Wu et al., 2005). *Chukrasia tabularis* A. Juss., an economically important evergreen tree, is widely cultured in tropical areas like Malaysia, southeastern China, and India (Luo et al., 2012). Its bark is traditionally used in China and India as astringent, antidiarrheal, and anti‐influenza agent, and its leaf extract exhibits activity on bacteria and fungi (Luo et al., 2010). Previous chemical research on this plant provided a series of phragmalin limonins. Limonin is a kind of nortriterpene with diversified structure, which has a wide range of bioactivities, like antifeeding insects, antimalarial and anticancer activities (Liao et al., 2009; Tan & Luo, 2011; Fang et al., 2011).

In the physiologic responses to infection or damage, macrophages have a special impact on the progress of inflammatory processes (Alivernini et al., 2020). Both the production of pro‐inflammatory mediator and the aggravation of inflammation are impossible to separate from the action of macrophage (Eissa et al., 2018). Many pro‐inflammatory cytokines, like tumor necrosis factor alpha (TNF‐*α*), interleukin 1*β* (IL‐1*β*), and interleukin 6 (IL‐6), are originated from macrophages. Nuclear factor kappa B (NF‐*κ*B) is an example of signal transduction and gene modulation associated with macrophages’ immersion and activation (Sae‐Tan et al., 2020). When activated, NF‐*κ*B causes the production of pro‐inflammatory cytokines like TNF‐*α*, IL‐6, and IL‐1*β*. Given the potential relevance of inflammation and macrophages, it is important to find a way to modulate the expression of inflammatory cytokines and control the activation of macrophages. Lipopolysaccharide (LPS) has been widely used to stimulate macrophages in inflammatory models in experiments on anti‐inflammatory mechanisms. After LPS stimulation, NF‐*κ*B signaling cascade was activated, resulting in changes in related protein expression (Ren et al., 2020).

**FIGURE 1:**
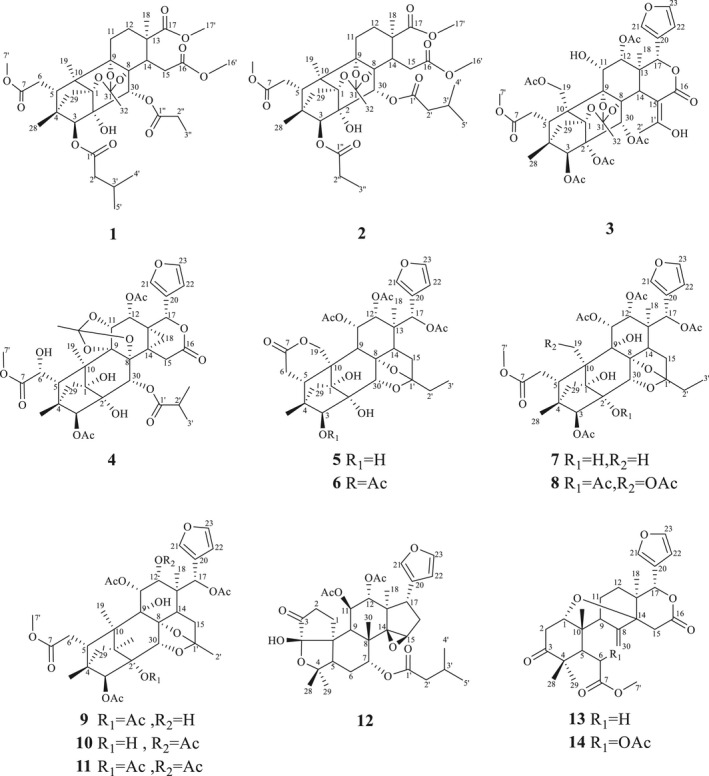
Chemical structures of compounds 1–14

In recent years, the anti‐inflammatory, antitumor, and antioxidant activities of *Chukrasia tabularis* have been widely reported (Kaur et al., 2011). In our studies on the anti‐inflammatory constituents of Meliaceae plants, two new phragmalin limonoid orthoesters Chukrasitin D and E (**1** and **2**) (Figure 1) were isolated and identified from the root barks of *C. tabularis*, together with 12 known limonoids (**3**–**14**). In this study, we report the separation, structure identification, and bioassay results of the extracts and isolated compounds. The in vitro anti‐inflammatory assay of compounds **1**–**14** on LPS‐mediated macrophages showed that limonins **1** and **2** displayed a significant inhibitory effect. In addition, the effects of limonin **1** on the production of nitric oxide, NF‐*κ*B, and TNF‐α in RAW 264.7 cells induced by LPS and their possible anti‐inflammatory mechanisms were also evaluated. Therefore, the current study focused on anti‐inflammatory evaluation of *C. tabularis* extracts and isolated limonins.

## 2 MATERIALS AND METHODS

### 2.1 Reagents and materials

The optical rotation was obtained using a JASCO P‐1020 polarimeter. Infrared (IR) spectra were measured on a Nicolet 170SX FT‐IR spectrometer, ultraviolet (UV) spectra were detected on a 210A UV spectrum. The nuclear magnetic resonance (NMR) spectra were recorded on a 400 MHz Bruker spectrometer. Electrospray ionization mass spectrometry (ESIMS) and high‐resolution electrospray ionization mass spectrometry (HRESIMS) were measured on a 2020 LCMS spectrum and Bruker APEX II mass spectrum, respectively. Semipreparative high‐performance liquid chromatography (HPLC) was performed on a RP‐18 column (250 × 10 mm, Waters). The root bark of *Chukrasia tabularis* was provided and identified by Dr. Y. H. Zhang, School of Pharmacy, Fujian Medical University, Fuzhou, China. Dulbecco’s modified Eagle’s medium (DMEM) was offered by Gibco Company (USA). The enzyme immunoassay kit of NF‐*κ*B, TNF‐*α*, and IL‐6 was supplied by the R&D System Company (USA). Lipopolysaccharide (LPS) was provided by Sigma Chemical Corp. (USA). Nitric oxide was supplied by Nanjing Jiancheng Bioengineering Inc. The electronic circular dichroism (ECD) spectrum was measured in methanol (MeOH) on a Jasco J 1500 spectropolarimeter (JASCO Corporation).

### 2.2 Preparation of extracts from *C. tabularis* and bioassay‐guided separation

The anti‐inflammatory test of xylene‐induced ear edema in mice showed that the dichloromethane extract had significant anti‐inflammatory activity (Table 1), so the dichloromethane phase was selected for further separation. Subfractions of dichloromethane extracts Fr.C and Fr.D showed significant anti‐inflammatory activity by mouse xylene auricle swelling experiments (Table 1), so isolation and purification focused on these two fractions.

**TABLE 1:** Inhibitory effect of *Chukrasia tabularis* root barks on ear edema induced by xylene in mice

Extracts/Fractions	Dose (mg/kg)	Edema degree ($\overset{-}{x}$ ± SD, mg)	Inhibition rate (%)
Control group		6.71 ± 0.33	
Aspirin	200	4.13 ± 0.96^*^	38.45
Methanol extract	400	5.12 ± 0.77^*^	38.50
Petroleum ether fraction	400	5.47 ± 0.81^*^	24.22
Dichloromethane fraction	400	4.39 ± 0.52^*^	42.41
Ethyl acetate fraction	400	5.31 ± 0.69^*^	31.89
n‐Butanol fraction	400	5.69 ± 0.46^*^	19.21
Fr. B(30%MeOH)	400	5.98 ± 0.83^*^	12.83
Fr. C(50%MeOH)	400	4.10 ± 0.64^*^	43.65
Fr. D(70%MeOH)	400	4.95 ± 0.85^*^	42.93
Fr. E(90%MeOH)	400	5.83 ± 0.79^*^	17.78

^*^ *p* < .01 versus Control group.

The chipped root bark of *C. tabularis* (5.6 kg) was extracted three times with MeOH at room temperature for 7 days each (20 L). The obtained solution was evaporated in vacuo to gain a brownish extract (890 g). The residue was suspended in H_2_O and divided by petroleum ether (PE), dichloromethane (CH_2_Cl_2_), ethyl acetate, and *n*‐butanol. The CH_2_Cl_2_ extract (290 g) was fractionated by a MCI gel column and eluted by 10% (Fr.A), 30% (Fr.B), 50% (Fr.C), 70% (Fr.D), 90% (Fr.E), and 100%MeOH (Fr.F). Fr.C (98 g) was fractionated to silica gel column and eluted by petroleum ether–ethyl acetate (PE–EtOAc) (8:1–0:1, each 5 L) to gain seven fractions (Frs. C1–C7). Fr.C3 (22.7 g) was fractionated to Sephadex LH‐20 (CH_2_Cl_2_‐MeOH) to give 6 fractions (Frs. C3‐1–C3‐6). Fr.C3‐2 (12.8 g) was applied to semi‐HPLC (MeCN:H_2_O = 7:3) to gain compounds **12** (15.6 mg, t_R_13.2 min), **4** (25.9 mg, t_R_17.1 min), **3** (21.8 mg, t_R_20.4 min), and **9** (15.4 mg, t_R_24.9 min). Fr.C3‐4 (10.2 g) was purified to HPLC (MeCN:H_2_O = 3:2) to obtain compounds **13** (12.5 mg, t_R_19.3 min), **14** (14.6 mg, t_R_21.9 min), **8** (37.6 mg, t_R_24.1 min), and **5** (27.4 mg, t_R_32.7 min). Fr.D (78 g) was fractionated with silica gel column and eluted by PE–EtOAc (8:1–0:1, each 5 L) to gain nine fractions (Frs. D1–D9). Fr.D3 (26.7 g) was applied to Sephadex LH‐20 (MeOH) to gain four fractions (Frs. D3‐1–D3‐4). Fr.D3‐1 (11.7 g) was subjected to RP‐18 column and semi‐HPLC (MeCN:H_2_O = 13:7) to gain limonins **6** (24.3 mg, t_R_17.2 min) and **11** (23.4 mg, t_R_22.6 min). Fr.D5 (13.8 g) was applied to Sephadex LH‐20 (MeOH) and semi‐HPLC (MeCN:H_2_O = 13:7) for compounds **1** (21.3 mg, t_R_15.1 min), **2** (13.7 mg, t_R_16.5 min), **7** (17.8 mg, t_R_24.5 min), and **10** (19.7 mg, t_R_33.7 min) (Figure S17).

### 2.3 Laboratory animals

Male Institute of Cancer Research (ICR) mice (18 ± 2 g), specific pathogen free, were supplied by the experimental animal center of Fujian Medical University. All animals were acclimatized to environment for 3 days before experiment, and fed and drank ad libitum. The animal experiments complied with the guidelines for the care and use of laboratory animals and were approved by the Laboratory Animal Ethics Committee of Fujian Medical University.

### 2.4 Xylene‐induced ear edema in mice

The extracts were dissolved in 0.5% CMC‐Na (sodium carboxymethyl cellulose) and Aspirin was applied as a positive control. After gavage of the extracts or control for 1 h, the right ear of each mouse was treated with 40 *μ*l of xylene solution, and the left ear served as a control. One hour after xylene treatment, mouse was executed due to cervical dislocation. A circular part with a diameter of 6 mm of each ear was weighed with an electronic analytical balance, and its inhibitory activity on ear edema was calculated (Table 1).

### 2.5 In vitro anti‐inflammatory activities

RAW 264.7 cells obtained from the China Center for Cultivated Studies (Shanghai, China) were maintained in DMEM contained with 1% penicillin and streptomycin and 10% fetal bovine serum, and under 5% CO_2_ at 37°C. Cells were stimulated with LPS. In brief, cells were placed on the 96‐well plate (1 × 10^5^ cells/well). After 2 h of preincubation, the LPS (2 *μ*g/ml) and compounds were added and the samples incubated for 24 h. The supernatant of cell culture was collected 24 h later and NO was detected by the Griess reagent (Gasparotto et al., 2013).

### 2.6 Measurement of NF‐κB, IL‐6, and TNF‐α production

The levels of NF‐κB, TNF‐α, and IL‐6 were determined by enzyme‐linked immunosorbent assay (ELISA) based on manufacturer's protocol. The standard solution and the antibody‐bearing sample were placed at 37°C for 60 min, added to the working solution, incubated in 37°C for 30 min, and washed. Tetramethylbenzidine (TMB) was then added and the TMB termination solution was added after 20 min. In the end, the absorbance at 450 nm was recorded by ELISA.

### 2.7 Statistical analysis

The data obtained were expressed as mean ± SD. All experiments had 3 replicates. The *t*‐test was used to verify differences between groups by IBM SPSS Statistics 24.

## 3 RESULTS

### 3.1 Bioactivity‐guided abstraction and isolation of active components

The anti‐inflammatory activities of methanolic, petroleum ether, dichloromethane (CH_2_Cl_2_), EtOAc, and *n*‐butanol extracts and fractions from the root barks of *C. tabularis* were assessed in vivo by xylene‐induced ear edema in mice. The result showed that the dichloromethane extract displayed significant anti‐inflammatory activities with an inhibition rate of 42.41% (400 mg/kg) (Table 1). The subfractions of dichloromethane extract Fr.C and Fr.D exhibited significant anti‐inflammatory activities with inhibitory values of 43.65% and 42.93% (400 mg/kg). Two novel phragmalin limonins, Chukrasitin D (**1**) and E (**2**), together with 12 known limonins (**3**–**14**) were separated and identified from Fr.C and Fr.D (Figure 1).

### 3.2 Structural elucidation of isolated compounds

Chukrasitin D (**1**) was isolated as white amorphous powder, and its molecular formula was demonstrated as C_35_H_50_O_14_ by the HRESIMS ion at *m*/*z* 718.3162 [M + Na + H]^+^ (calcd. for C_35_H_51_O_14_Na, 718.3175) which indicated 11 degrees of unsaturation. The infrared (IR) spectrum analysis indicated that **1** contained hydroxyl (3459 cm^−1^) and ester groups (1740 cm^−1^). The ^13^CNMR (carbon nuclear magnetic resonance) spectrum indicated 35 carbon resonances, including 10 methyl groups (three methoxys), seven methylene groups, five methane groups (two oxygenated), and 13 quaternary carbons (five oxygenated). In addition, a comprehensive analysis of its ^1^HNMR (proton nuclear magnetic resonance) and ^13^CNMR (carbon nuclear magnetic resonance) and data (Table 2) showed the presence of three methyl esters, one orthoacetate moiety, one propanoyl, and one 3‐methylbutyryl group. In molecule **1**, there are 11 unsaturates, of which 5 are occupied by 5 ester carbonyls, and the remaining 6 unsaturates require **1** to be hexacyclic in the center. The foregoing data indicated that **1** was a limonoid orthoester of phragmalin type (Lin et al., 2009).

**TABLE 2:** ^1^H‐NMR (proton nuclear magnetic resonance) (400 MHz) and ^13^C‐NMR (carbon nuclear magnetic resonance) (100 MHz) spectroscopic data for 1 and 2

No.	1^a^		2^a^	
	*δ*_H_ (*J* in Hz)	*δ*_C_	*δ*_H_ (*J* in Hz)	*δ*_C_
1		87.3		88.3
2		81.0		80.9
3	4.54 (s)	85.6	4.55 (s)	85.4
4		46.7		46.6
5	2.94 (brd, 11.7)	37.8	2.85 (m)	38.0
6	2.49 (dd, 13.5, 6.0)	35.7	2.51 (m)	35.6
	2.93 (dd, 13.5, 6.0)		2.90 (dd, 11.9, 5.3)	
7		174.7		175.3
8		87.2		87.0
9		88.5		87.2
10		47.3		47.1
11*α*	1.28 (m)	29.4	1.30 (m)	30.3
11*β*	1.32 (m)		1.35 (m)	
12*α*	1.33 (m)	26.1	1.19 (m)	26.1
12*β*	1.38 (m)		1.23 (m)	
13		35.5		35.3
14	2.19 (m)	44.0	2.17 (m)	44.2
15	2.78 (dd, 12.3, 7.0)	26.3	2.28 (dd, 12.4, 5.0)	27.2
	3.18 (brd, 11.7)		2.77 (m)	
16		171.2		171.8
17		174.8		174.8
18	1.14 (s)	20.7	1.13 (s)	20.7
19	1.18 (s)	16.6	1.18 (s)	16.7
28	0.95 (s)	15.2	0.91 (s)	15.0
29a	1.98 (d, 13.0)	40.5	1.65 (d, 10.7)	40.5
29b	1.66 (d, 13.0)		1.96 (d, 10.7)	
30	5.90 (s)	72.6	5.81 (s)	72.6
31		120.4		120.5
32	1.62 (s)	21.3	1.62 (s)	21.3
1´		179.3		179.2
2´	2.33 (m)	28.7	2.35 (m)	28.7
3´	2.93 (m)	35.4	2.75 (m)	34.9
4´	1.24 (d, 7.2)	18.7	1.14 (d, 6.5)	18.7
5´	1.17 (d, 7.2)	18.7	1.19 (d, 6.5)	18.7
1′´		174.1		174.3
2′´	2.30 (q, 8.0)	28.7	2.36 (q, 7.5)	28.7
3′´	1.03 (t, 8.0)	9.0	1.04 (t, 7.5)	9.0
7´‐OMe	3.73 (s)	52.8	3.73 (s)	52.8
16´‐OMe	3.73 (s)	52.8	3.73 (s)	52.8
17´‐OMe	3.73 (s)	52.8	3.73 (s)	52.8

^a^ Recorded in CD_3_OD.

Extensive 2DNMR (two‐dimensional nuclear magnetic resonance) spectral analysis, especially HMBC (heteronuclear multiple bond correlation) data, assigned most of the functional units to limonoid core and identified D‐Seco Phragmalin limonoid framework of **1**. In HMBC spectra, the main correlation between H_3_‐18/ C‐13/ C‐12/ C‐17 and between an OMe to C‐17 at *δ*174.8 and the correlation between H_2_‐15/OMe of C‐16 *δ*171.2 indicated that the two OMe units were connected, respectively, to C‐17 and C‐16 (Figure S1), suggesting that **1** was ring D‐ring‐opened limonin (Lin et al., 2009). The mutual HMBC correlations of H‐3 and C‐3'of 3‐methylbutyryloxy moiety showed that it is situated in C‐3. The HMBC correlation between H‐30 and carbonyl (C‐1′′) of a propanoyl group *δ*174.1 exhibited the presence of a propanoyloxy in C‐30. The quaternary carbon *δ*120.4 (C‐31) corresponds to methyl *δ*1.62(s), showing the presence of orthoacetate part. In addition, the strong HMBC correlation between C‐1/H‐29/H‐19, C‐2/H‐29/H‐3, C‐18/H‐30/H‐15, and C‐9/H‐12/H‐11 allocated four oxygenated quaternary carbons as C‐2, C‐1, C‐9, and C‐8. Phragmalin limonoids, separated from *Chukrasia*, are usually 8, 9, 14‐, 8, 9, 30‐, and 1, 8, 9‐ orthoacetate (Lin et al., 2011; Silva et al., 2008; Zhang, Fan, et al., 2008; Zhang, Yang, et al., 2008). The presence of 1, 8, 9 orthoacetate in **1** was determined temporarily.

**FIGURE 2:**
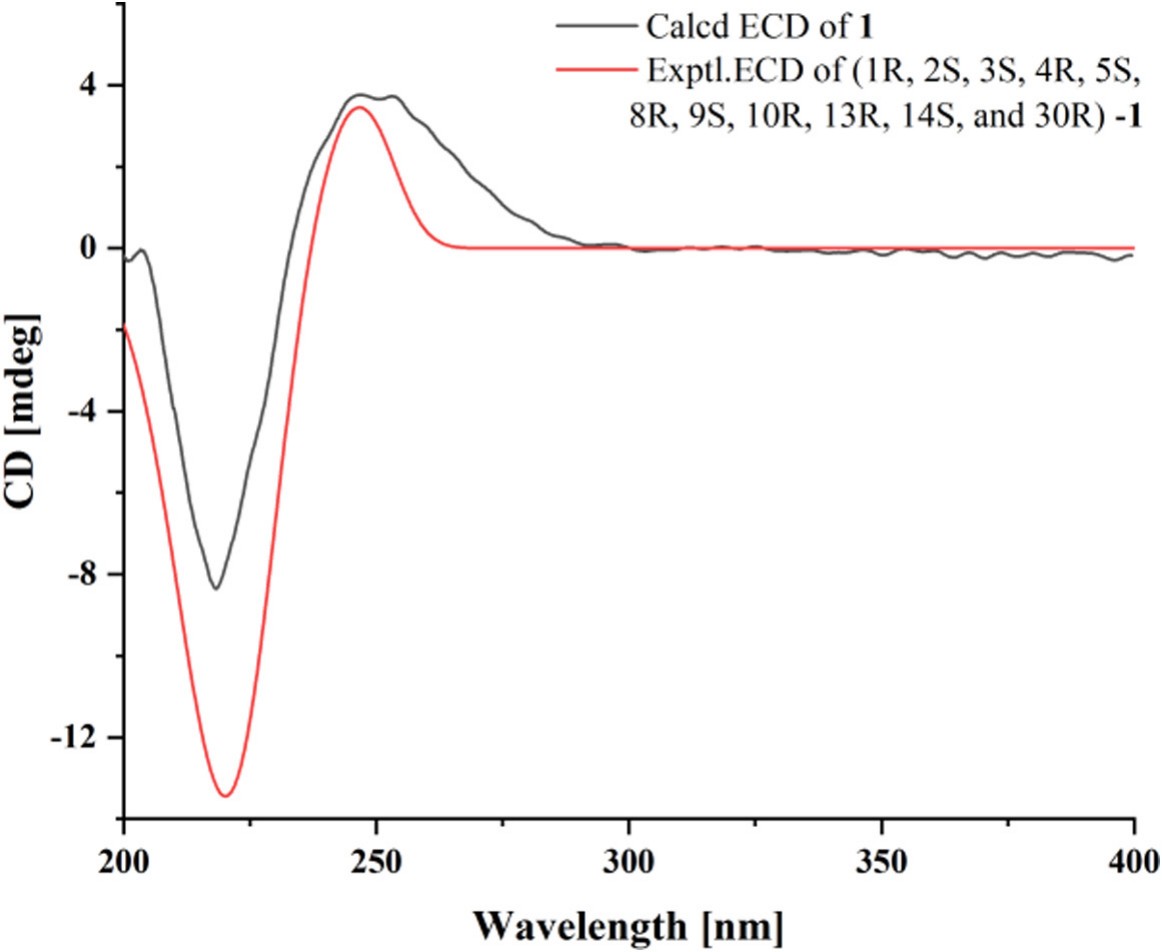
Calculated and experimental electronic circular dichroism (ECD) spectra of 1

The relative structure of compound **1** was deduced by ROESY (Rotating Frame Overhauser Enhancement Spectroscopy) spectra, and there were strong cross peaks between Me‐18*α/*H‐14 and H‐30/H‐15, which showed that H‐30 and H‐15 were coplanar, and they were *β* directed. The ROESY correlation of H‐3/H‐29 and Me‐4′/H‐5 exhibited that the 3‐methylbutyryl moiety was *β* oriented. The main correlation of H‐18/H‐14 revealed that H‐14 was *α* directed. H‐32 is associated with methyl of propanoyl and H‐14, indicating that propanoyl and 1, 8, 9 orthoacetate groups were *α* directed. The ROESY relationship between H‐29a/H‐19/Me‐28 and between H‐29b/Me‐28/H‐3 can determine the two protons of C‐29. According to the above results, the relative configuration of **1** was completed, as shown in Figure 1. By comparing experiments and computational ECD data, the absolute configuration of **1** was finally proved, which is a suitable method for solving the absolute configuration of natural products (Michalska et al., 2017; Zhao et al., 2018). ECD spectra were calculated by systematic conformational search, geometric optimization, and time‐dependent density functional theory calculations using Gaussian‐16 software. The calculated ECD spectra are in good agreement with the experimental spectra, pointing to the absolute configuration **1** of 1*R*, 2*S*, 3*S*, 4*R*, 5*S*, 8*R*, 9*S*, 10*R*, 13*R*, 14*S*, and 30*R* (Figure 2). Thus, the stereochemistry of **1** was constructed as shown in Figure 1 (Figure S1).

Chukrasitin E (**2**) was found to possess a quasimolecular ion peak [M + Na + K]^+^ at *m*/*z* 756.2718 (calcd 756.2753) in HRESIMS, and its molecular formula was demonstrated as C_35_H_50_O_14_, which was the same as compound 1. The IR and mass spectrometry (MS) spectrum of 1 and 2 are almost the same, and their ^1^H and ^13^CNMR data also indicate high similarity and differences by the chemical shifts of H‐3 *δ* 4.54 for 1 and *δ* 4.55 for 2, and H‐30 *δ5.90* for **1** and *δ*5.81 for **2** (Table 2), indicating that **2** may be the 3, 30‐isomer of 1. In its HMBC spectra, the strong correlation of H‐3 with the carbonyl (C‐1′′) of a propanoyl at *δ* 174.3 indicated the presence of a propanoyloxy in C‐3. The HMBC correlation of H‐30 with C‐1′ of the 3‐methylbutyryloxy moiety showed that it is located at C‐30. The relative structure of **2** was deduced by ROESY spectra and there were strong cross peaks between Me‐18*α/*H‐14 and H‐30/H‐15, which showed that H‐30 and H‐15 were coplanar, and they were *β* directed. The ROESY correlation of H‐3/H‐29 and Me‐3′′/H‐5 exhibited that the propanoyl moiety was *β* oriented. The main correlation of H‐18/H‐14 revealed that H‐14 was *α* directed. H‐32 is associated with methyl of 3‐methylbutyryl and H‐14, indicating that 3‐methylbutyryl and 1, 8, 9 orthoacetate groups were *α* directed. The ROESY relationship between H‐29a/H‐19/Me‐28 and between H‐29b/Me‐28/H‐3 can determine the two protons of C‐29. Thus, the stereochemistry of **2** was constructed as shown in Figure 1 (Figure S9).

In addition to limonins **1** and **2**, 12 known limonins, namely Velutinasin A (**3**) (Zhang et al., 2014), Tabularisin J (**4**) (Zhang, Fan, et al., 2008; Zhang, Yang, et al., 2008), Chuktabularin H (**5**) (Luo et al., 2010), Chuktabularin E (**6**) (Luo et al., 2010), Chuktabularin Q (7) (Luo et al., 2010), Chuktabularin L (**8**) (Luo et al., 2012), Chuktabularin T (**9**) (Luo et al., 2010), Chuktabularin S (**10**) (Luo et al., 2010), Chuktabularin A (**11**) (Zhang, Yang, Liao, et al., 2007; Zhang, Yang, Zhu, et al., 2007), dumsin (**12**) (Nihei et al., 2004), methyl angolensate (**13**) (Mireku et al., 2014), and 6‐acetoxy (**14**) (Abdelgaleil et al., 2001), have been isolated and their structures were confirmed by previously reported data (Figure 1).

#### 3.2.1 NMR and ESI‐MS spectroscopic data

Chukrasitin D (**1**): white powder; [*α*]^28^_D_: −0.68° (c 0.36, CHCl_3_); UV (MeOH)209 nm; IR (KBr): ν_max_ 3459, 2970, 1740, 1406, 1150, 1043 cm^−1^; ^1^H and ^13^C NMR data, see Table 2; HRESIMS *m/z* 718.3162 [M + Na + H]^+^ (calcd. For C_35_H_51_O_14_Na, 718.3175).

Chukrasitin E (**2**): white powder; [*α*]^28^_D_: −0.66° (*c* 0.30, CHCl_3_); UV (MeOH)209 nm; IR (KBr): ν_max_ 3450, 2971, 1742, 1460, 1151, 1047 cm^−1^; ^1^H and ^13^C NMR data, see Table 2; HRESIMS *m/z* 756.2718 [M + Na + K]^+^ (calcd. 756.2753).

### 3.3 Anti‐inflammatory effects of separated limonins from *C. tabularis*

Nitric oxide is a major bioinformatics molecule with dual roles of biological messenger and cytotoxic molecule. It is involved in the pathogenicity of inflammation, is overexpressed in LPS‐mediated macrophages, and is an indicator of inflammation (Jeon et al., 2016; Keisuke et al., 2021). The in vitro anti‐inflammatory effects of limonins (**1**–**14**) were determined by LPS‐stimulated RAW 264.7 cells by evaluating the production of NO. Cell viability determination showed that limonins (**1**–**14**) have no toxicity to RAW 264.7 cells at a concentration of 100 *μ*M. To determine whether limonins (**1**–**14**) suppressed NO production by LPS‐mediated RAW 264.7 cells, the concentration of NO in medium containing these limonins was evaluated. As shown in Table 3, 14 limonins exhibited anti‐inflammatory effects at the tested concentration. The result exhibited that D‐ring‐opened phragmalin limonoid orthoester (**1**–**2**) showed strong NO inhibitory activities, while limonins (**3**–**14**) showed potent to moderate activity. Limonins **1**–**2** displayed significant anti‐inflammatory activities with IC_50_ values of 6.24 and 6.13 μM. Limonoids **3**–**14** showed effective anti‐inflammatory effect with the inhibition rate between 12.30 and 50.19 *μ*M. Considering that anti‐inflammatory components are found in the root bark of *C. tabularis*, it can be determined that they are a source of natural anti‐inflammatory molecules. It is worth noting that limonins **1** and **2** showed the strongest anti‐inflammatory activity. Therefore, the potential anti‐inflammatory activity and molecular mechanism of compound **1** were further studied.

**TABLE 3:** Inhibitory activity of compounds 1–14 on lipopolysaccharide‐induced (LPS‐induced) nitric oxide (NO) production in RAW 264.7 cells

Compounds	IC_50_(*μ*M)	Compounds	IC_50_(*μ*M)
1	6.24 ± 0.80	**9**	31.79 ± 7.11
2	6.13 ± 0.99	**10**	36.78 ± 3.66
**3**	25.56 ± 3.21	**11**	27.83 ± 1.11
**4**	21.78 ± 4.2	**12**	50.19 ± 11.78
**5**	12.30 ± 4.21	**13**	48.25 ± 5.38
6	20.46 ± 6.38	**14**	44.95 ± 7.74
7	25.76 ± 2.12	**Indomethacin** ^a^	26.18 ± 1.56
**8**	19.86 ± 3.90		

^**a**^ Positive control.

### 3.4 Effects of limonin **1** on LPS‐stimulated production of TNF‐*α*, IL‐6, and NF‐*κ*B

**FIGURE 3:**
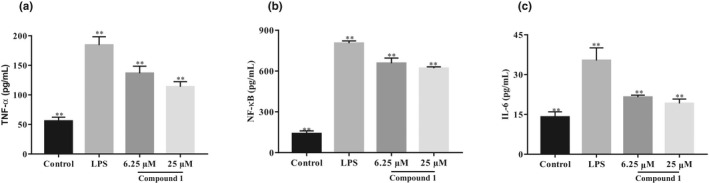
Effects of compound 1 on lipopolysaccharide (LPS)‐induced macrophages. The macrophages were incubated with LPS (1 μg/ml) and treated with compound 1 (6.25 and 25 μM) for 24 h. (a)The levels of tumor necrosis factor alpha (TNF‐α), (b) nuclear factor kappa B (NF‐κB), and (c) interleukin 6 (IL‐6) in the supernatant were assayed using enzyme‐linked immunosorbent assay (ELISA) kits. All values are the mean ± SD. **p < .01 compared to the LPS group

TNF‐*α*, IL‐6, and NF‐*κ*B are major pro‐inflammatory cytokines identified as anti‐inflammatory markers that can be released by LPS‐mediated macrophages (Dong et al., 2018; Lee et al., 2021). Excessive production of pro‐inflammatory cytokines exacerbates a variety of diseases, including allergies, autoimmune disease, and cancer (Benedetto et al., 2019; Guo et al., 2019). We investigated the activity of limonin **1** on LPS‐stimulated pro‐inflammatory cytokines in RAW 264.7 cells. The results in Figure 3 exhibited that the levels of NF‐*κ*B, IL‐6, and TNF‐*α* in the LPS group were notably higher than those in the control group. As shown in Figure 3a–c, the addition of limonin **1** notably suppressed production of NF‐κB, IL‐6, and TNF‐α in a dose‐dependent manner. The result indicated that limonin **1** could suppress the expression of NF‐κB, IL‐6, and TNF‐α in LPS‐stimulated macrophage, and achieved anti‐inflammatory activity. The regulation of anti‐inflammatory activity on macrophages may be partly involved in the protective activity of *Chukrasia tabularis* on inflammatory diseases.

## 4 DISCUSSION

Macrophage is involved in most inflammatory responses, including LPS stimulation, and secretes pro‐inflammatory cytokines like NF‐κB, TNF‐α, and IL‐6 (Kim et al., 2020). Modern studies have suggested that natural product may inhibit inflammation by modulating NO or inflammatory factor in macrophage (Fang et al., 2008). Among these natural products, a thorough in‐depth research on homologous medicinal and edible plants, it is found that limonin is the main anti‐inflammatory active ingredient (Fan et al., 2019), mainly through inhibition of inflammatory mediators’ NF‐κβ signaling cascade (Jin et al., 2018). *C. tabularis* bark and fruit extract was proven to have anti‐inflammatory activities by inhibiting pro‐inflammatory cytokines such as NO, TNF‐α, and IL‐6 (Perianayagam et al., 2004). However, few reports have focused on anti‐inflammatory activities and mechanism of limonins in *C. tabularis* extracts (Yang et al., 2020). In this study, a combination of octadecyl silica gel, Sephadex LH‐20, and HPLC was used to separate anti‐inflammatory limonins from *C. tabularis*, and 14 compounds were identified by NMR, ECD, and mass spectrometry, including two novel limonins Chukrasitin D and E (**1** and **2**) and 12 known limonins (**3**–**14**). Further research revealed that limonins **1**–**2** exhibited notable anti‐inflammatory activities by LPS‐mediated RAW 264.7 cells. Furthermore, limonin **1** can suppress the release of TNF‐α, NF‐κB, and IL‐6 stimulated by LPS, thereby contributing to the anti‐inflammatory activity of the extract.

Lipopolysaccharide is a common pathogenic endotoxin constituent of the outer membrane of Gram‐negative bacteria. LPS is an effective trigger for inflammatory responses (Pandher et al., 2021). Inflammation is the main risk element for many diseases, and macrophages are the primary immune cells and the first line of defense against pathogen invasion (Leseigneur et al., 2020). In the process of inflammation, macrophages produce excess induced nitric oxide synthase as inflammatory mediators and pro‐inflammatory cytokines such as TNF‐α, IL‐6, and IL‐1β (Huang et al., 2019). NO is a biological signal and effect or element that modulates the expression of pro‐inflammatory cytokine (Zamora et al., 2000). Inflammatory damage is thought to be caused by the excessive production of NO‐induced pro‐inflammatory cytokine (Kany et al., 2019; Zhang et al., 2017). Excessive production of nitric oxide occurs in inflammation and various diseases, where NO plays a cytotoxic role in the pathological process (Lea et al., 2020; Shao et al., 2013). Therefore, suppression of NO production is important for the prevention of inflammatory disease. Among inflammatory stimulants, LPS induces macrophage activation leading to the release of pro‐inflammatory cytokine in the inflammatory response (Bonizzi & Karin, 2004; Ronchetti et al., 2017). Cytokines arouse fever, stun, and various inflammatory diseases. Therefore, it is essential to suppress the overproduction of pro‐inflammatory cytokines. The in vitro anti‐inflammatory effects of limonins (**1**–**14**) were determined by LPS‐stimulated RAW 264.7 cells by evaluating the production of NO. The result exhibited that D‐ring‐opened phragmalin limonoid orthoester (**1**–**2**) showed strong NO inhibitory activities while limonins (**3**–**14**) showed potent to moderate activity. We also investigated the activity of limonin **1** on LPS‐stimulated pro‐inflammatory cytokines in RAW 264.7 macrophages. The results showed that limonin **1** notably inhibited the expression of NF‐κB, IL‐6, and TNF‐α in LPS‐stimulated macrophage, and achieved anti‐inflammatory activity. The regulation of anti‐inflammatory activity on macrophages may be partly involved in the protective activity of *Chukrasia tabularis* on inflammatory diseases.

Our results showed that fractions of *C. tabularis* root barks’ ethanol extract indicated varying degrees of anti‐inflammatory effect on ear swelling induced by xylene in mice. Two isolated anti‐inflammatory limonins, Chukrasitin D (**1**) and Chukrasitin E (**2**), exhibited remarkable inhibitory activity. The result showed that limonin **1** suppressed the production of NO and pro‐inflammatory cytokines in LPS‐stimulated RAW 264.7 cells in a dose‐dependent manner. This is the first time that Chukrasitin D (**1**) and Chukrasitin E (**2**) have been identified in *C. tabularis* root bark, and they have been proved to have strong anti‐inflammatory activities, providing a theoretical basis for the application of *C. tabularis* in anti‐inflammatory activity.

## 5 CONCLUSIONS

Screening for anti‐inflammatory activity of *Chukrasia tabularis* root bark extracts led to the separation of 14 limonins, including two novel phragmalin limonoids (**1**–**2**), 12 known limonoids (**3**–**14**). All isolated compounds were determined for NO production by LPS‐mediated RAW 264.7 cells. The result exhibited that D‐ring‐opened phragmalin limonoid orthoester (**1**–**2**) showed strong NO inhibitory activities while limonins (**3**–**14**) showed potent to moderate activity. Limonins **1**–**2** displayed significant anti‐inflammatory activities with IC_50_ values of 6.24 and 6.13 μM. Compound 1 inhibited the production of NO and TNF‐α in stimulated cells and reduced the secretion of NO and TNF‐α levels during inflammatory processes. These results provide basic information for further research on utilizing *C. tabularis* as natural anti‐inflammatory resource.

## CONFLICT OF INTEREST

The authors declare no conflicts of interest.

## DATA AVAILABILITY STATEMENT.

The data presented in this study are available in the Supplementary Materials.

## ETHICAL APPROVAL

All experiments involving the use of animals have been approved by the Institutional Animal Protection and Use Committee of Fujian Medical University (Approval No. 2017‐0102).

## Supporting information


Figures S1–S17

## References

[fsn33015-bib-0001] Abdelgaleil, S. A. M. , Doe, M. , Morimoto, Y. , & Nakatani, M. (2006). Phragmalin limonoids from *Chukrasia tabularis* . Phytochemistry, 65(20), 2833–2841. 10.1002/chin.200511185 15474570

[fsn33015-bib-0002] Abdelgaleil, S. A. M. , Okamura, H. , Iwagawa, T. , Sato, A. , Miyahara, I. , Doe, M. , & Nakatani Khayanolides, M. (2001). Rearranged phragmalin limonoid antifeedants from *Khaya senegalensis* . Tetrahedron, 57(1), 119–126. 10.1016/s0040–4020(00)00994–7

[fsn33015-bib-0003] Alivernini, S. , MacDonald, L. , Elmesmari, A. , Finlay, S. , Tolusso, B. , Gigante, M. R. , Petricca, L. , Di Mario, C. , Bui, L. , Perniola, S. , Attar, M. , Gessi, M. , Fedele, A. L. , Chilaka, S. , Somma, D. , Sansom, S. N. , Filer, A. , McSharry, C. , Millar, N. L. , … Kurowska‐Stolarska, M. (2020). Distinct synovial tissue macrophage subsets regulate inflammation and remission in rheumatoid arthritis. Nature Medicine, 26, 1295–1306. 10.1038/s41591-020-0939-8 32601335

[fsn33015-bib-0004] Benedetto, P. D. , Ruscitti, P. , Vadasz, Z. , Toubi, E. , & Giacomelli, R. (2019). Macrophages with regulatory functions, a possible new therapeutic perspective in autoimmune diseases. Autoimmunity Reviews, 18(10), 102369. 10.1016/j.autrev.2019.102369 31404701

[fsn33015-bib-0005] Bonizzi, G. , & Karin, M. (2004). The two NF‐kB activation pathways and their role in innate and adaptive immunity. Trends in Immunology, 25(6), 280–288. 10.1016/j.it.2004.03.008 15145317

[fsn33015-bib-0006] Dong, J. , Li, J. , Cui, L. , Wang, Y. , Lin, J. , Qu, Y. , & Wang, H. (2018). Cortisol modulates inflammatory responses in LPS‐stimulated RAW264.7 cells via the NF‐κB and MAPK pathways. BMC Veterinary Research, 14(30), 1–10. 10.1186/s12917-018-1360-0 29378573 PMC5789647

[fsn33015-bib-0007] Eissa, N. , Hussein, H. , Kermarrec, L. , Ali, A. Y. , Marshall, A. , Metz‐Boutigue, M. , Hendy, G. N. , Bernstein, C. N. , & Ghia, J. E. (2018). Chro‐mogranin‐A regulates macrophage function and the apoptotic pathway in murine DSS colitis. Journal of Molecular Medicine, 96, 183–198. 10.1007/s00109-017-1613-6 29274006

[fsn33015-bib-0008] Fan, C. Q. , Wang, X. N. , Yin, S. , Zhang, C. R. , Wang, F. D. , & Yue, J. M. (2007). Tabularisins A‐D, phragmalin ortho esters with new skeleton isolated from the seeds of *Chukrasia tabularis* . Tetrahedron, 63, 6741–6747. 10.1016/j.tet.2007.04.078

[fsn33015-bib-0009] Fan, S. M. , Zhang, C. L. , Luo, T. , Wang, J. Q. , Tang, Y. , Chen, Z. M. , & Yu, L. Y. (2019). Limonin: A review of its pharmacology, toxicity, and pharmacokinetics. Molecules, 24, 3679–3701. 10.3390/molecules24203679 31614806 PMC6832453

[fsn33015-bib-0010] Fang, S. , Rao, Y. K. , & Zheng, Y. M. (2008). Anti‐oxidant and inflammatory mediator's growth inhibitory effects of compounds isolated from *Phyllanthus urinaria* . Journal of Ethnopharmacology, 116, 333–340. 10.1016/j.jep.2007.11.040 18187278

[fsn33015-bib-0011] Fang, X. , Di, Y. T. , & Hao, X. J. (2011). The advances in the limonoid chemistry of the Meliaceae family. Current Organic Chemistry, 15(9), 1363–1391. 10.2174/138527211795378254

[fsn33015-bib-0012] Gasparotto, J. , Bittencourt Pasquali, M. A. , Somensi, N. , Vasques, L. M. , Moreira, J. , Claudio, F. , Almeida, R. N. , Barbosa‐Filho, J. M. , Fátima, V. S. M. , Gutierrez, S. J. C. , Júnior, L. J. Q. , & Gelain, D. P. (2013). Effect of *N*‐salicyloyltryptamine (STP), a novel tryptamine analogue, on parameters of cell viability, oxidative stress, and immunomodulation in RAW 264.7 macrophages. Cell Biology and Toxicology, 21(4), 175–187. 10.1007/s10565-013-9245-2 23605514

[fsn33015-bib-0013] Guo, X. , Cheng, L. , Yang, S. , & Che, H. (2019). Pro‐inflammatory immunological effects of adipose tissue and risk of food allergy in obesity: Focus on immunological mechanisms. Allergologia et Immunopathologia, 48(3), 306–312. 10.1016/j.aller.2019.06.004 31477390

[fsn33015-bib-0014] Huang, J. , Ning, N. , & Zhang, W. W. (2019). Effects of paraquat on IL‐6 and TNF‐α in macrophages. Experimental and Therapeutic Medicine, 17(3), 1783–1789. 10.3892/etm.2018.7099 30783450 PMC6364147

[fsn33015-bib-0015] Jeon, H. L. , Yoo, J. M. , Lee, B. D. , Lee, S. J. , Sohn, E. J. , & Kim, M. R. (2016). Anti‐inflammatory and antioxidant actions of N‐Arachidonoyl serotonin in RAW264.7 Cells. Pharmacology, 97, 195–206. 10.1159/000443739 26859139

[fsn33015-bib-0016] Jin, S. W. , Wang, J. Q. , Chen, S. Y. , Jiang, A. D. , Jiang, M. L. , Su, Y. R. , Yan, W. , Xu, Y. G. , & Gong, G. Q. (2018). A novel limonin derivate modulates inflammatory response by suppressing the TLR4/NF‐κB signalling pathway. Biomedicine & Pharmacotherapy, 100, 501–508. 10.1016/j.biopha.2018.02.046 29477914

[fsn33015-bib-0017] Kany, S. W. , Vollrath, J. T. , & Relja, B. (2019). Cytokines in inflammatory disease. International Journal of Molecular Sciences, 20(23), 6008. 10.3390/ijms20236008 31795299 PMC6929211

[fsn33015-bib-0018] Kaur, R. , & Arora, S. (2009). Chemical constituents and biological activities of *Chukrasia tabularis* A. Juss.‐A review. Journal of Medicinal Plant Research, 3(4), 196–216. 10.1089/jmf.2008.0009

[fsn33015-bib-0019] Kaur, R. , Sharma, U. , Singh, B. , & Arora, S. (2011). Antimutagenic and antioxidant characteristics of *Chukrasia tabularis* A juss extracts. International Journal of Toxicology, 30(1), 21–34. 10.1177/1091581810385362 20959614

[fsn33015-bib-0020] Keisuke, S. , Ryosuke, T. , & Koji, W. (2021). Epalrestat suppresses inflammatory response in lipopolysaccharide‐stimulated RAW264.7 cells. Allergologia et Immunopathologia, 49(5), 1–8. 10.15586/aei.v49i5.102 34476915

[fsn33015-bib-0021] Kim, M. Y. , Kim, H. J. , Lee, Y. Y. , Kim, M. H. , Lee, J. Y. , Kang, M. S. , Koo, B. C. , & Lee, B. W. (2020). Antioxidant and anti‐inflammatory effects of Peanut (*Arachis hypogaea* L.) skin extracts of various cultivars in oxidative‐damaged HepG2 cells and LPS‐induced raw 264.7 macrophages. Food Science & Nutrition, 9(4), 1–12. 10.1002/fsn3.2064 PMC7866586 33598180

[fsn33015-bib-0022] Lea, W. , Angela, S. , & Markus, N. (2020). Bioengineering of anti‐inflammatory natural products. ChemMedChem, 16(5), 767–776. 10.1002/cmdc.202000771 33210441 PMC7986114

[fsn33015-bib-0023] Lee, J. , Son, W. , Hong, J. , Song, Y. , Yang, C. S. , & Kim, Y. H. (2021). Down‐regulation of TNF‐α via macrophage‐targeted RNAi system for the treatment of acute inflammatory sepsis. Journal of Controlled Release, 336, 344–353. 10.1016/j.jconrel.2021.06.022 34147573

[fsn33015-bib-0024] Leseigneur, C. , Lê‐Bury, P. , Pizarro‐Cerdá, J. , & Dussurget, O. (2020). Emerging evasion mechanisms of macrophage defenses by pathogenic bacteria. Frontiers in Cellular and Infection Microbiology, 10, 577559. 10.3389/fcimb.2020.577559 33102257 PMC7545029

[fsn33015-bib-0025] Liao, S. G. , Chen, H. D. , & Yue, J. M. (2009). Plant Orthoesters. Chemical Reviews, 109, 1092–1140. 10.1021/cr0782832 19182998

[fsn33015-bib-0026] Lin, B. D. , Zhang, C. R. , Yang, S. P. , Wu, Y. , & Yue, J. M. (2011). Phragmalin‐type limonoid orthoesters from the twigs of *Swietenia macrophylla* . Chemical & Pharmaceutical Bulletin, 59(4), 458–465. 10.1248/cpb.59.458 21467674

[fsn33015-bib-0027] Lin, B. D. , Zhang, C. R. , Yang, S. P. , Zhang, S. , Wu, Y. , & Yue, J. M. (2009). D‐ring‐opened phragmalin‐type limonoid orthoesters from the twigs of *Swietenia macrophylla* . Journal of Natural Products, 42(37), 1305–1313. 10.1002/chin.201137192 19552385

[fsn33015-bib-0028] Luo, J. , Li, Y. , Wang, J. S. , Lu, J. , Wang, X. B. , Luo, J. G. , & Kong, L. Y. (2012). Twelve novel and diverse 16‐norphragmalin‐type limonoids from *Chukrasia tabularis var. velutina* . Chemical & Pharmaceutical Bulletin, 60(2), 195–204. 10.1002/chin.201232204 22293478

[fsn33015-bib-0029] Luo, J. , Wang, J. S. , Wang, X. B. , Luo, J. G. , & Kong, L. Y. (2010). Chuktabularins E‐T, 16‐norphragmalin limonoids from *chukrasia tabularis var. velutina* . Journal of Natural Products, 73(5), 835–843. 10.1021/np900734c 20405929

[fsn33015-bib-0030] Michalska, K. , Bednarek, E. , Gruba, E. , Lewandowska, K. , Mizera, M. , & Cielecka‐Piontek, J. (2017). Comprehensive spectral identification of key intermediates to the final product of the chiral pool synthesis of radezolid. Chemistry Central Journal, 11(82), 1–16. 10.1186/s13065-017-0309-x 29086862 PMC5549672

[fsn33015-bib-0031] Mireku, E. A. , Mensah, A. Y. , Mensah, M. L. K. , Tochher, D. A. , & Habtemariam, S. (2014). Antiinflammatory properties of the stem‐bark of *Anopyxis klaineana* and its major constituent, methyl angolensate. Phytotherapy Research, 28(12), 1855–1860. 10.1002/ptr.5212 25111951

[fsn33015-bib-0032] Mulholland, D. A. , Parel, B. , & Coombes, P. H. (2000). The chemistry of the meliaceae and ptaeroxylaceae of southern and eastern Africa and Madagascar. Current Organic Chemistry, 4, 1011–1054. 10.2174/1385272003375941

[fsn33015-bib-0033] Nakatani, M. , Abdelgaleil, S. A. M. , Saad, M. M. G. , Huang, R. C. , Doe, M. , & Iwagawa, T. (2004). Phragmalin limonoids from *Chukrasia tabularis* . Phytochemistry, 65(20), 2833–2841. 10.1002/chin.200511185 15474570

[fsn33015-bib-0034] Nihei, K. I. , Asaka, Y. , Mine, Y. , Ito, C. , Furukawa, H. , Juichi, M. , & Kubo, I. (2004). Insect antifeedants from tropical plants: Structures of dumnin and dumsenin. Journal of agricultural and food chemistry, 52, 3325–3328. 10.1021/jf049819c 15161191

[fsn33015-bib-0035] Pandher, U. , Kirychuk, S. , Schneberger, D. , Thompson, B. , Aulakh, G. , Sethi, R. S. , & Singh, B. (2021). Pulmonary inflammatory response from co‐exposure to LPS and glyphosate. Environmental Toxicology and Pharmacology, 86, 103651. 10.1016/j.etap.2021.103651 33812014

[fsn33015-bib-0036] Perianayagam, J. B. , Sharma, S. K. , Joseph, A. , & Christina, A. (2004). Evaluation of anti‐pyretic and analgesic activity of Emblica officina lis Gaertn. Journal of Ethnopharmacology, 95(1), 83–85. 10.1016/j.jep.2004.06.020 15374611

[fsn33015-bib-0037] Ren, J. , Su, D. , Li, L. , Cai, H. , Zhang, M. , Zhai, J. , Li, M. , Wu, X. , & Hu, K. (2020). Anti‐inflammatory effects of aureusidin in lps‐stimulated raw264. 7 macrophages via suppressing NF‐*κ*B and activating ros‐and mapks‐dependent Nrf2/HO‐1 signaling pathways. Toxicology and Applied Pharmacology, 387, 114846. 10.1016/j.taap.2019.114846 31790703

[fsn33015-bib-0038] Ronchetti, S. , Migliorati, G. , & Delfino, D. V. (2017). Association of inflammatory mediators with pain perception. Biomedicine & Pharmacotherapy, 96, 1445–1452. 10.1016/j.biopha.2017.12.001 29217162

[fsn33015-bib-0039] Roy, A. , & Saraf, S. (2006). Limonoids: Overview of significant bioactive triterpenes distributed in plants kingdom. Biological & Pharmaceutical Bulletin, 29(2), 191–201. 10.1248/bpb.29.191 16462017

[fsn33015-bib-0040] Saad, M. M. G. , Iwagawa, T. , Doe, M. , & Nakatani, M. (2003). Swietenialides, novel ring D opened phragmalin limonoid orthoesters from *Swietenia mahogani* Jacq. Tetrahedron, 59, 8027–8033. 10.1016/j.tet.2003.08.033

[fsn33015-bib-0041] Sae‐Tan, S. , Kumrungsee, T. , & Yanaka, N. (2020). Mungbean seed coat water extract inhibits inflammation in LPS‐induced acute liver injury mice and LPS‐stimulated RAW 246.7 macrophages via the inhibition of TAK1/I*κ*B*α*/NF‐*κ*B. Journal of Food Science and Technology, 57, 2659–2668. 10.1007/s13197-020-04302-y 32549616 PMC7270297

[fsn33015-bib-0042] Shao, J. , Li, Y. , Wang, Z. , Xiao, M. , Yin, P. , Lu, Y. , Qian, X. , Xu, Y. , & Liu, J. (2013). A novel naphthalimide derivative, exhibited anti‐inflammatory effects via targeted‐inhibiting TAK1 following down‐regulation of ERK1/2‐ and p38 MAPK‐mediated activation of NF‐κB in LPS‐stimulated RAW264.7 macrophages. International Immunopharmacology, 17(2), 216–228. 10.1016/j.intimp.2013.06.00 23810444

[fsn33015-bib-0043] Silva, M. N. , Arruda, M. S. P. , Castro, K. C. F. , Silva, M. G. F. , Fernandes, J. B. , & Vieira, P. C. (2008). Limonoids of the phragmalin type from *Swietenia macrophylla* and their chemotaxonomic significance. Journal of Natural Products, 71, 1983–1987. 10.1021/np800312h 19053509

[fsn33015-bib-0044] Tan, Q. G. , & Luo, X. D. (2011). Meliaceous limonoids: Chemistry and biological activities. Chemical Reviews, 111, 7437–7522. 10.1021/cr9004023 21894902

[fsn33015-bib-0045] Wu, J. , Xiao, Q. , Zhang, S. , Li, X. , Xiao, Z. H. , Ding, H. X. , & Li, Q. X. (2005). Xyloccensins Q‐V, six new 8, 9, 30‐phragmalin ortho ester antifeedants from the Chinese mangrove *Xylocarpus granatum* . Tetrahedron, 61, 8382–8389. 10.1016/j.tet.2005.06.099

[fsn33015-bib-0046] Yang, R. , Song, C. , Chen, J. , Zhou, L. , Jiang, X. , Cao, X. , & Zhang, Q. (2020). Limonin ameliorates acetaminophen‐induced hepatotoxicity by activating Nrf2 antioxidative pathway and inhibiting NF‐κB inflammatory response via upregulating Sirt1. Phytomedicine, 69, 153211. 10.1016/j.phymed.2020.153211 32259676

[fsn33015-bib-0047] Zamora, R. , Vodovotz, Y. , & Billiar, T. R. (2000). Inducible nitric oxide synthase and inflammatory diseases. Molecular Medicine, 6(5), 347–373. 10.1007/bf03401781 10952018 PMC1949959

[fsn33015-bib-0048] Zhang, C. R. , Fan, C. Q. , Zhang, L. , Yang, S. P. , Wu, Y. , Lu, Y. , & Yue, J. M. (2008). Chuktabrins A and B, two novel limonoids from the twigs and leaves of *Chukrasia tabularis* . Organic Letters, 15, 3183–3186. 10.1021/ol800885h 18605730

[fsn33015-bib-0049] Zhang, C. R. , Yang, S. P. , Chen, X. Q. , Wu, Y. , Zhen, X. C. , & Yue, J. M. (2008). Limonoids from the twigs and leaves of *Chukrasia tabularis* . Helvetica Chimica Acta, 91, 2338–2350. 10.1002/hlca.200890254

[fsn33015-bib-0050] Zhang, C. R. , Yang, S. P. , Liao, S. G. , Fan, C. Q. , Wu, Y. , & Yue, J. M. (2007). Chuktabularins A‐D, four new limonoids with unprecedented carbon skeletons from the stem bark of *Chukrasia tabularis* . Organic Letters, 9(17), 3383–3386. 10.1021/ol701437h 17650011

[fsn33015-bib-0051] Zhang, C. R. , Yang, S. P. , Zhu, Q. , Liao, S. G. , Wu, Y. , & Yue, J. M. (2007). Nortriterpenoids from *Chukrasia tabularis var. velutina* . Journal of Natural Products, 70(10), 1616–1619. 10.1021/np070345w 17929895

[fsn33015-bib-0052] Zhang, F. , Zhang, C. R. , Tao, X. , Wang, J. , Chen, W. S. , & Yue, J. M. (2014). Phragmalin‐type limonoids with NF‐κB inhibition from *chukrasia tabularis var. velutina* . Bioorganic & Medicinal Chemistry Letters, 24(16), 3791–3796. 10.1016/j.bmcl.2014.06.069 25037915

[fsn33015-bib-0053] Zhang, Q. S. , Heng, Y. , Yuan, Y. H. , & Chen, N. H. (2017). Pathological α‐synuclein exacerbates the progression of Parkinson's disease through microglial activation. Toxicology Letters, 265, 30–37. 10.1016/j.toxlet.2016.11.002 27865851

[fsn33015-bib-0054] Zhao, S. , Yan, X. , Zhao, Y. , Wen, J. , Zhao, Z. , & Liu, H. (2018). Dihydroisocoumarins from *Radix Glycyrrhizae* . BMC Chemistry, 12(58), 1–6. 10.1186/s13065-018-0427-0 PMC5945569 29748827

