# Supplementary material for: Bioactivity‐guided isolation of anti‐inflammatory limonins from Chukrasia tabularis
Source: Food Sci Nutr. 2022 Aug 9;10(12):4216–25. doi: 10.1002/fsn3.3015 (PMC9731525; doi:10.1002/fsn3.3015)
Supplement: Supplementary file 1 — Figures S1–S17 [file FSN3-10-4216-s001.docx]

**Bioactivity-guided isolation of anti-inflammatory limonins from *Chukrasia tabularis***

Jin-huang Shen^1^, Yi-fan Zhang ^2^, Li Zhang^1^, Na-na Yang^1^, Xin-hua Ma^1^, Tian-hua Zhong^3^ and Yong-hong Zhang^1^

^1^Fujian Provincial Key Laboratory of Natural Medicine Pharmacology, School of Pharmacy, Fujian Medical University, Fuzhou, China

^2^Medical Imaging Department, First Affiliated Hospital of Fujian Medical University, Fuzhou, China;

^3^Key Laboratory of Marine Biogenetic Resources, Third Institute of Oceanography, Ministry of Natural Resources, Xiamen, China

**Correspondence**

Yong-hong Zhang, School of Pharmacy, Fujian Medical University, Fuzhou 350122, China. Email: zhangyh@fjmu.edu.cn

Tian-hua Zhong, Third Institute of Oceanography, Ministry of Natural Resources, Xiamen 361005, China. Email: zhongtianhua@tio.org.cn

**Bioactivity-guided isolation of anti-inflammatory limonins from *Chukrasia tabularis***

**Abstract:** *Chukrasia tabularis* is an economically important tree and widely cultured in the southeast of China. Its barks, leaves and fruits are consumed as a traditional medicine and perceived as a valuable source for bioactive limonin compounds. The extracts from root barks of *C. tabularis* showed significant anti-inflammatory effect. The aim of this research is to explore the material basis of *C. tabularis* anti-inflammatory activity, and to purify and identify anti-inflammatory active ingredients. By a bioassay-guided isolation of dichloromethane fraction obtained two novel phragmalin limonins, Chukrasitin D and E (**1** and **2**), together with twelve known limonins (**3-14**). The chemical structure of these compounds is determined on the basis of extensive spectral analysis and chemical reactivity. In addition, the activities of these isolated limonins on production of NO, TNF-α, and NF-*κ*B in RAW264.7 cells induced by LPS were evaluated. Limonins **1** and **2** indicated significant anti-inflammatory activity with IC_50_ values of 6.24 and 6.13*μ*M. Compound **1** notably inhibited the production of NF-*κ*B, TNF-*α* and IL-6 in macrophages*.* The present results suggest that the root barks of *C. tabularis* exhibited anti-inflammatory effect and the limonins may be responsible for the activity.

**Keywords:** Chukrasia tabularis; limonins; NO; TNF-*α*; anti-inflammation

**List of Supporting Information**

**Figure S1**. Key ^1^H-^1^H COSY, HMBC and NOESY correlations of compound **1.**

**Figure S2**. ^1^H NMR spectrum (400 MHz) of compound **1** in CD_3_OD.

**Figure S3**. ^13^C NMR spectrum (100 MHz) of compound **1** in CD_3_OD.

**Figure S4.** HMBC spectrum of compound **1** in CD_3_OD.

**Figure S5.** HSQC spectrum of compound **1** in CD_3_OD.

**Figure S6**. ^1^H-^1^H COSY spectrum of compound **1** in CD_3_OD.

**Figure S7**. ^1^H-^1^H NOESY spectrum of compound **1** in CD_3_OD.

**Figure S8**. HRESIMS spectrum of compound **1**.

**Figure S9**. Key ^1^H-^1^H COSY, HMBC and NOESY correlations of compound **2.**

**Figure S10**. ^1^H NMR spectrum (400 MHz) of compound **2** in CD_3_OD.

**Figure S11**. ^13^C NMR spectrum (100 MHz) of compound **2** in CD_3_OD.

**Figure S12**. HMBC spectrum of compound **2** in CD_3_OD.

**Figure S13.** HSQC spectrum of compound **2** in CD_3_OD.

**Figure S14.** ^1^H-^1^H COSY spectrum of compound **2** in CD_3_OD.

**Figure S15**. ^1^H-^1^H NOESY spectrum of compound **2** in CD_3_OD.

**Figure S16**. HRESIMS spectrum of compound **2**.

**Figure S17.** HPLC analysis of Fr.C3-3, Fr.C3-4, Fr.D3-1 and Fr.D5 (at 210 nm).

**Figure S1.** Key ^1^H-^1^H COSY, HMBC and NOESY correlations of compound **1.**

**Figure S2**. ^1^H NMR spectrum (400 MHz) of compound **1** in CD_3_OD.

**Figure S3**. ^13^C NMR spectrum (100 MHz) of compound **1** in CD_3_OD.

**Figure S4**. HMBC spectrum of compound **1** in CD_3_OD.

**Figure S5**. HSQC spectrum of compound **1** in CD_3_OD.

**Figure S6**. ^1^H-^1^H COSY spectrum of compound **1** in CD_3_OD.

**Figure S7**. ^1^H-^1^H NOESY spectrum of compound **1** in CD_3_OD.

**
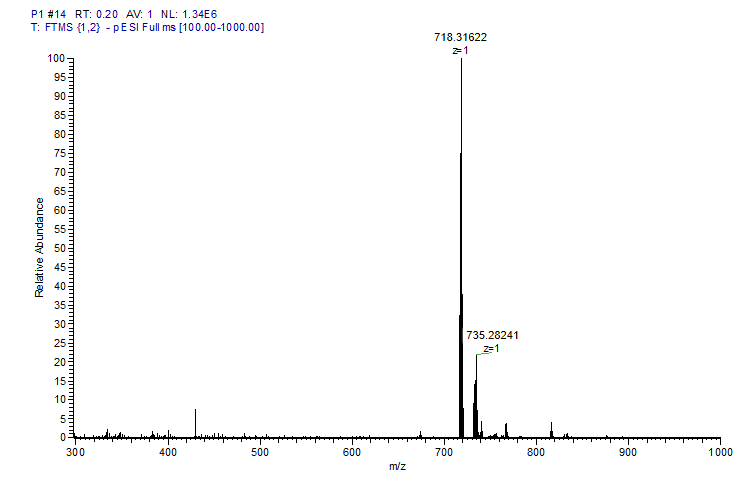
**

**Figure S8**. HRESIMS spectrum of compound **1**.

**Figure S9.** Key ^1^H-^1^H COSY, HMBC and NOESY correlations of compound **2.**

**Figure S10**. ^1^H NMR spectrum (400 MHz) of compound **2** in CD_3_OD.

**Figure S11**. ^13^C NMR spectrum (100 MHz) of compound **2** in CD_3_OD.

**Figure S12**. HMBC spectrum of compound **2** in CD_3_OD.

**Figure S13**. HSQC spectrum of compound **2** in CD_3_OD.

**Figure S14**. ^1^H-^1^H COSY spectrum of compound **2** in CD_3_OD.

**Figure S15**. ^1^H-^1^H NOESY spectrum of compound **2** in CD_3_OD.

**
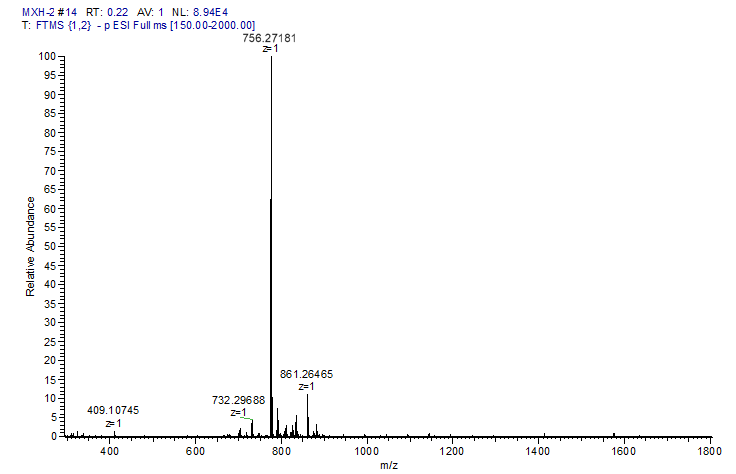
**

**Figure S16**. HRESIMS spectrum of compound **2**.

9

3

4

12

Fr.C3-3

5

8

14

13

Fr.C3-4

11

6

Fr.D3-1

10

7

2

1

Fr.D5

**Figure S17.** HPLC analysis of Fr.C3-3, Fr.C3-4, Fr.D3-1 and Fr.D5 (at 210 nm).

12
